# Vesicoamniotic shunting for fetal megacystis in the first trimester with a Somatex^®^ intrauterine shunt

**DOI:** 10.1007/s00404-020-05598-z

**Published:** 2020-05-24

**Authors:** B. Strizek, I. Gottschalk, F. Recker, E. Weber, A. Flöck, U. Gembruch, A. Geipel, C. Berg

**Affiliations:** 1 Department of Obstetrics and Prenatal Medicine, University Hospital Bonn, Venusberg-Campus 1, 53127 Bonn, Germany; 2 Division of Prenatal Medicine, Department of Obstetrics and Gynecology, University Hospital Cologne, Cologne, Germany

**Keywords:** LUTO, Megacystis, Vesicoamniotic shunt, Posterior urethral valve

## Abstract

**Purpose:**

The objective was to evaluate the feasibility of vesicoamniotic shunting (VAS) in the first trimester with the Somatex^®^ intrauterine shunt and report on complications and neonatal outcome.

**Methods:**

Retrospective cohort study of all VAS before 14 weeks at two tertiary fetal medicine centres from 2015 to 2018 using a Somatex^®^ intrauterine shunt. All patients with a first trimester diagnosis of megacystis in male fetuses with a longitudinal bladder diameter of at least 15 mm were offered VAS. All patients that opted for VAS after counselling by prenatal medicine specialists, neonatologists and pediatric nephrologists were included in the study. Charts were reviewed for complications, obstetric and neonatal outcomes.

**Results:**

Ten VAS were performed during the study period in male fetuses at a median GA of 13.3 (12.6–13.9) weeks. There were two terminations of pregnancy (TOP) due to additional malformations and one IUFD. Overall there were four shunt dislocations (40%); three of those between 25–30 weeks GA.

Seven neonates were born alive at a median GA of 35.1 weeks (31.0–38.9). There was one neonatal death due to pulmonary hypoplasia. Neonatal kidney function was normal in the six neonates surviving the neonatal period. After exclusion of TOP, perinatal survival was 75%, and 85.7% if only live-born children were considered.

**Conclusion:**

VAS in the first trimester is feasible with the Somatex^®^ Intrauterine shunt with low fetal and maternal complication rates. Neonatal survival rates are high due to a reduction in pulmonary hypoplasia and the rate of renal failure at birth is very low. VAS can be safely offered from the late first trimester using the Somatex^®^ intrauterine shunt.

## Introduction

Urinary tract anomalies, although being among the most frequent congenital anomalies diagnosed prenatally, are very rare. Obstructive uropathy has a prevalence of 3 in 10,000 at birth and megacystis has been reported to have a prevalence of 1:1345 in a first trimester screening population [1]. An enlarged bladder (megacystis) is one of the key findings in lower urinary tract obstruction (LUTO), but there are other non-obstructive pathologies leading to fetal megacystis. The most common etiology in male fetuses is posterior urethral valves (PUV), followed by urethral stenosis or atresia. Depending on the degree and time of onset of the obstruction and oligohydramnios, respectively, mortality due to lung hypoplasia is high (45–100%) and elevated pressure in the urinary tract and kidneys leads to renal dysplasia and insufficiency of varying degrees in survivors. 60–80% of megacystis are nowadays diagnosed in the first trimester [2]. Its diagnosis has been reported to be reliable if the longitudinal bladder diameter (LBD) is > 15 mm [1] and detection in the first trimester in itself seems to carry a worse prognosis compared to second or third trimester diagnosis [3–5]. Pulmonary hypoplasia due to anhydramnios before 20 weeks is the main cause of neonatal mortality. Vesicoamniotic shunting (VAS) in the second trimester has been shown to reduce neonatal mortality by reducing pulmonary morbidity, but the effect on long-term renal function remains questionable. Even in cases with a first trimester diagnosis, VAS is often withheld until > 16 weeks of gestation due to technical difficulties with the previously used shunt systems, high complication rates, especially PPROM, as well as diagnostic challenges in the first trimester.

However, there is evidence from animal studies that time of onset and duration of bladder obstruction correlate with the degree of renal impairment [6–11], we therefore speculate that prenatal intervention aiming at not only lowering pulmonary hypoplasia, but also preserving renal function should be performed as early as possible, preferably before 14 weeks GA.

We have been offering vesicoamniotic shunting (VAS) from the first trimester with the Somatex^®^ Intrauterine Shunt from late 2014. In this study, we report on technical success, complication rates and perinatal outcome in VAS performed before 14 weeks of gestation.

## Material and methods

We conducted a retrospective cohort study in two tertiary fetal medicine centers.

Patients with a prenatal diagnosis of isolated megacystis in the first trimester and VAS with a Somatex^®^ intrauterine shunt not later than 13 + 6 weeks of gestation in male fetuses in a 4-year period (2015–2018) were included. Megacystis was defined as a longitudinal bladder diameter of > 15 mm in the mid-sagittal plane. A systematic sonographic evaluation was performed in all cases transabdominally, and transvaginally if necessary, including nuchal translucency measurement and early echocardiography.

VAS was offered in fetuses without additional malformations or suspicion of aneuploidy on ultrasound. Conventional karyotyping by CVS was recommended to all patients before VAS, but VAS was performed even if parents refused invasive testing. Parents were extensively counselled by fetal medicine specialists, neonatologists and pediatric nephrologists prior to VAS and all provided written informed consent. Vesicocentesis was not performed before VAS. Patients were followed weekly by ultrasound after shunt placement and VAS was repeated as soon as possible after dislocation occurred.

**Fig. 1:**
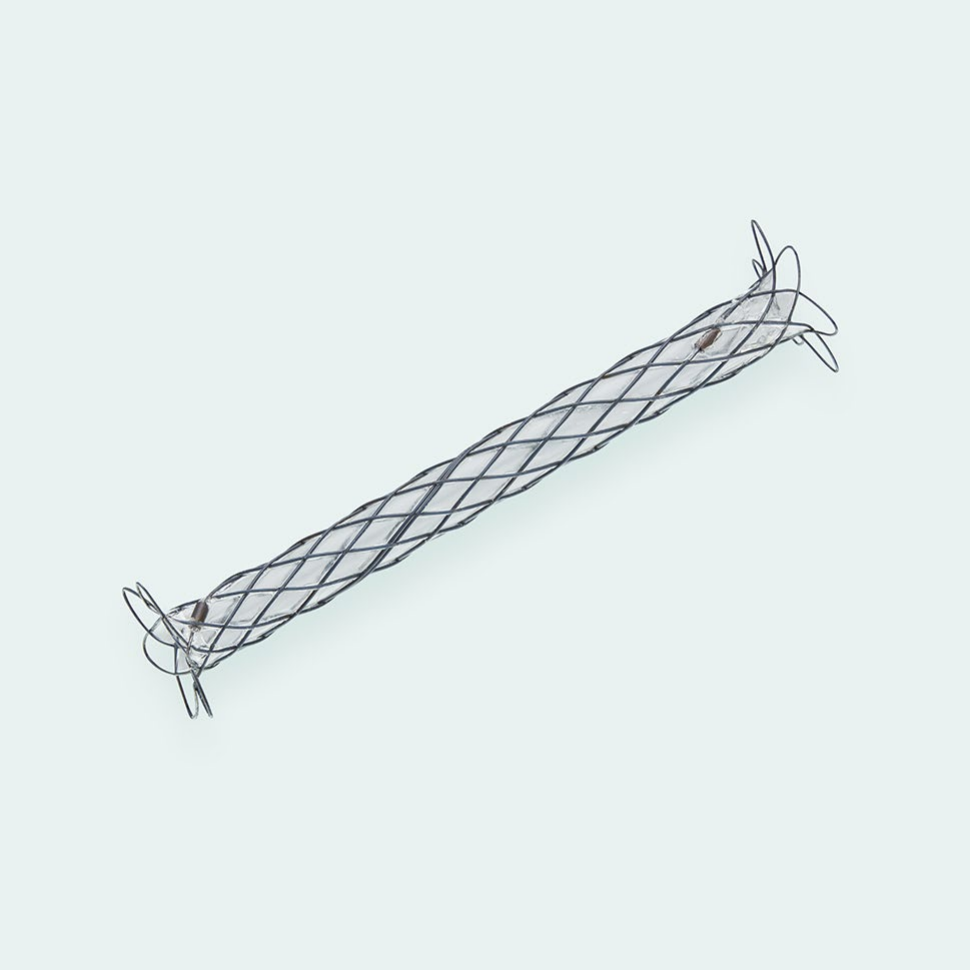
Somatex^®^ Intra Uterine Shunt. The shunt consisting of a nitinol wire mesh and impermeable silicone coating with silicone-free, self-deploying parasols at both ends of the shunt with two X-ray markers

The Somatex^®^ intrauterine shunt (Somatex Medical, Germany) is 22 mm long with a diameter of 1.2 mm consisting of a nitinol wire mesh and internal impermeable silicone coating. (Fig. 1) The shunt has self-deploying parasols at both ends and can be placed through an 18 G puncture cannula (outer diameter 1.27 mm) without the need for maternal or fetal anesthesia. The shunt is preloaded into the cannula, therefore it is not possible to acquire fetal urine samples during the procedure and amniotic fluid can only be sampled after shunt deployment.

Obstetric and pediatric charts were reviewed for associated anomalies, prenatal course of pregnancy, amount of amniotic fluid during pregnancy, prenatal and neonatal complications, neonatal outcome and interventions after birth.

Ethical approval was waived for retrospective archive studies by the ethics commitees of the universities of Bonn and Cologne. The data have been presented as part of a larger cohort as an oral presentation at a conference [12].

## Results

**Fig. 2:**
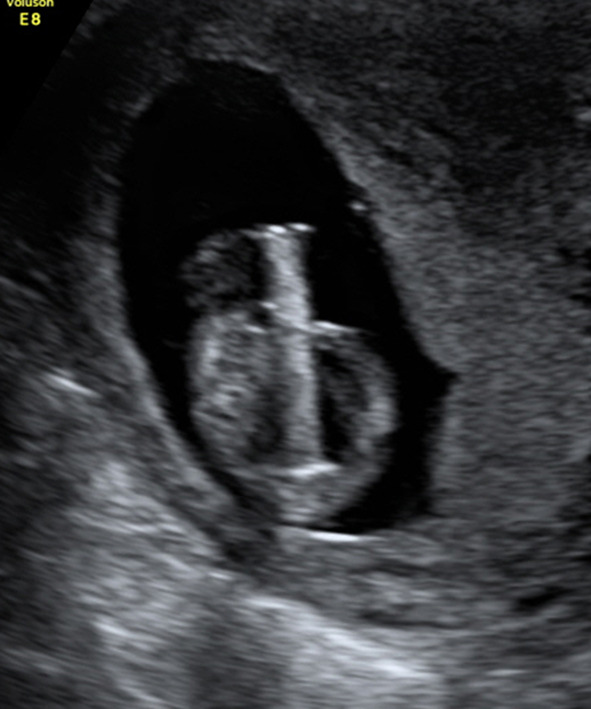
Correct shunt position in a transverse view after vesicoamniotic shunting at 12 + 4 weeks. The shunt can be easily visualized due to its nitinol wire mesh. The fetal bladder is empty

During the study period, ten patients underwent VAS before 14 weeks of gestation in our two centers. Shunt placement was technically successful in all ten patients (Fig. 2).

Median gestational age at diagnosis was 12.9 weeks, VAS was performed at a median age of 13.3 weeks of gestation (12.6–13.9 weeks). Median CRL was 71 (62–82 mm) at diagnosis. Nuchal translucency was > 95. Centile in four fetuses, all with normal karyotype. Amniotic fluid was normal in all cases at the time of the intervention and amnioninfusion was not necessary to perform VAS in any patient. Access was transplacental in eight cases.

Median longitudinal bladder diameter was 30 mm (21–36 mm). In 8/10 fetuses, there was sonographic evidence of a keyhole sign. In one fetus, there was evidence of spontaneous bladder rupture with urinary ascites, but the bladder was still enlarged (LBD 36 mm) on ultrasound. VAS was successfully performed after drainage of ascites.

The kidneys were considered abnormal in three cases (either hyperechogenic, presence of hydronephrosis or both), but without signs of renal dysplasia at the time of diagnosis. In four patients (40%), CVS for conventional karyotyping was performed prior to VAS, the remaining parents refused CVS. All surviving fetuses had a normal karyotype confirmed either pre- or postnatally.

### Intrauterine course and shunt complications

Initial shunt placement was technically successful in all ten cases. There was no rupture of membranes, bleeding or infection immediately following the procedure and no shunt-related maternal complications during the study period.

There was one IUFD at 17 + 4 weeks GA, approximately 4 weeks after the intervention without any other evident cause.

Two (20%) patients decided for termination of pregnancy (TOP) in the late second or third trimester after detection of severe additional malformations; in one fetus due to severe hydrocephalus with kyphoscoliosis at 20 weeks GA and in another due to severe hydrocephalus due to bilateral intracranial hemorrhage at 30 weeks GA. Both parents refused post-mortem examination. The amount of amniotic fluid was normal at the time of TOP in both pregnancies.

**Fig. 3:**
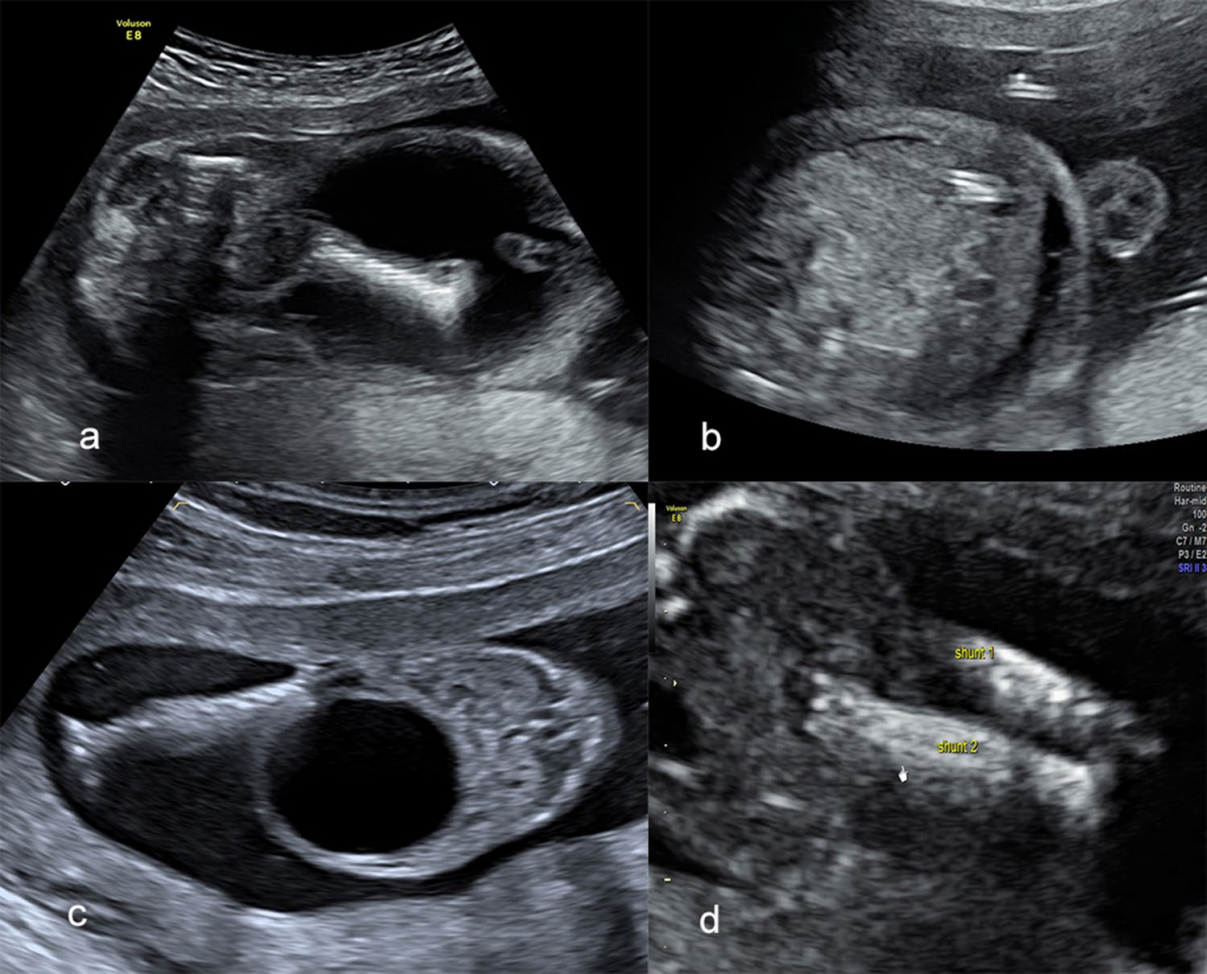
Images illustrating shunt dislocations. **a** Intraabdominal shunt dislocation. The distal end of the shunt (on the right side of the image) is dislocated into the abdominal cavity leading to urinary ascites. **b** After placement of an abdomino-amniotic Harrison shunt the ascites has decreased. **c** The proximal end of the shunt is dislocated subcutaneously. The bladder is still enlarged. **d** A second Somatex^®^ shunt (shunt 2) is correctly positioned in the fetal bladder, which is empty, shunt 1 can be seen next to shunt 2

Overall, there were four shunt dislocations (40%), none of which into the amniotic fluid (Fig. 3). In one fetus, the shunt dislocated subcutaneously into the abdominal wall after 11 days and a second shunt was placed successfully at 15 + 0 weeks. There were three shunt dislocations of the distal end of the shunt into the abdominal cavity of the fetus with development of urinary ascites at 12, 16 and 17 weeks, respectively after initial shunting. In those three cases, a second shunt was placed in the abdominal wall as a abdomino-amniotic shunt at 25, 29 and 30 weeks. Ascites disappeared in all three fetuses. In two live-born neonates, the dislocated shunt had to be removed by laparotomy, with the need of re-laparotomy in one due to paralytic ileus. In all other neonates, the shunt was removed without any problems.

Only in one case, there was anhydramnios from 24 weeks onwards, all other patients reaching the third trimester had normal amniotic fluid.

### Outcome

Seven out of ten (70%) fetuses were born alive at a median gestational age of 35.1 weeks (31–39 weeks). (Table 1) Route of delivery was caesarean section in two and vaginal delivery in five patients. Reason for delivery was PPROM/spontaneous onset of labour in five and non-reassuring CTG in one child the day following the insertion of a second abdomino-amniotic shunt. Premature rupture of membranes and/or delivery before 34 weeks occurred in three pregnancies (30%), including the one with a re-intervention the day before delivery. There was no delivery prior to 28 weeks and two prior to 32 weeks.

**Table 1:** Characteristics of ten patients receiving VAS in the first trimester

	GA @intervention, weeks	Sex	LBD (mm)	Re-intervention, weeks GA	GA at birth/TOP	Outcome	Diagnosis after birth	Neonatal renal function
1	13.9	m	31	30.9, AAS	31	Alive	PUV	Normal
2	13.1	m	21			IUFD (17 + 4 weeks)		
3	13.0	m	26		32	Alive	PUV, VUR	Normal
4	12.7	m	30		31	Alive	Urethral stenosis, patent urachus	Normal
5	12.7	m	36		20	TOP, normal amniotic fluid	Bilateral, symmetric hydrocephaly of unknown etiology, scoliosis	
6	13.4	m	31	25.4, AAS	30	TOP, normal amniotic fluid	intracranial hemorrhage, etiology unclear	
7	13.9	m	26	29.7, AAS	35	Alive	PUV, VUR	Normal
8	12.6	m	30		36	Alive	DSD 46, XY, cloacal dygenesis (agenesis of penis and urethra, anal atresia), recto-vesical fistula Muscular. VSD, ASD II, wedge vertebra	Normal
9	13.4	m	27	15 + 0, VAS	38	NND	Pulmonary hypoplasia, partial agenesis of corpus callosum, anal atresia, scrotum bifidum	Abnormal (microcystic, hypoplastic kidneys at postnatal ultrasound)
10	13.6	m	36 (afterbladder rupture)		38	Alive	Urethral stenosis, anal atresia with fistula	Normal

*ASD* atrial septal defect, *DSD* disorder of sexual development, *GA* gestational age, *IUFD* intrauterine fetal death, *LBD* longitudinal bladder diameter, *NND* neonatal death, *AAS* abdomino-amniotic shunt, *PUV* posterior urethral valves, *TOP* termination of pregnancy, *VAS* vesicoamniotic shunt, *VSD* ventricular septal defect, *VUR* vesicoureteral reflux, *m* male

**Fig. 4:**
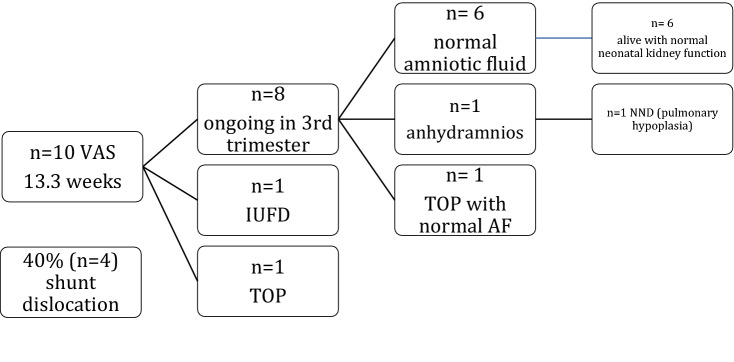
Outcome in 10 fetuses after first trimester VAS. *IUFD* intrauterine fetal death, *NND* neonatal death, *TOP* termination of pregnancy, *VAS* vesicoamniotic shunt

Neonatal survival among live-born children was 85.7% (6/7). Perinatal survival on an intention to treat calculation was 60% (6/10) and 75% after exclusion of TOP (Fig. 4). One neonate died of pulmonary hypoplasia during the first day of life. In this case, anhydramnios had been evident from 24 weeks despite a correctly positioned shunt with an empty bladder. Furthermore, the kidneys were hypoplastic with cystic changes on pre- and postnatal ultrasound. This neonate presented with additional suspicion of partial agenesis of the corpus callosum, anal atresia and scrotum bifidum. The parents refused a post-mortem examination.

Among the six surviving children only one prematurely born neonate had signs of mild pulmonary hypoplasia. Renal function was considered normal during the neonatal period in all surviving neonates by the treating neonatologists. In the neonate that died due to lung hypoplasia, intrauterine renal failure was suspected based on the sonografic findings of anhydramnios and microcystic kidneys.

### Postnatal diagnosis and associated malformations

In the six survivors, the underlying pathology was PUV in three (50%) and urethral stenosis in two (33.3%). One of the latter had additional high anal atresia, that was treated with a sigmoidostomy and the other had a patent urachus. The last boy has a disorder of sexual development (DSD 46, XY) that had been diagnosed prenatally after shunt placement with a complex cloacal malformation (cloacal dysgenesis) with a flat perineum with agenesis of urethra, penis and rectum treated with vesico- and descendostoma after birth.

## Discussion

We describe the first cohort of undergoing VAS before 14 weeks GA. We could demonstrate that VAS using a Somatex^®^ intrauterine shunt is technically feasible, as all shunts were placed successfully without the need of amnioninfusion. There was no miscarriage or early rupture of membranes and no maternal shunt-related complications, contrary to what has been reported in the literature in the few reported cases with early VAS using different, thicker types of shunts [13].

The introducer of the Somatex shunt is considerably smaller (18 G) than for the Harrison Fetal Bladder Stent (13 G/2.41 mm) (Cook Medical^®^) or the Rocket^®^KCH™ Fetal Bladder Drain (3 mm). The smaller size allowed early shunt placement and the design of the shunt led to low dislocation rates. Both of these factors contributed to a neonatal survival rate of live-born fetuses of 85.7% in our cohort with a very low incidence of pulmonary hypoplasia (1/7). This leads to a considerable reduction of neonatal mortality compared to untreated fetuses with megacystis in the first trimester or after second trimester VAS [14–16]. The live-birth rate after a first trimester diagnosis without VAS has been reported to be as low as 0–15% [4, 5, 17]. As in most other first trimester cohorts, mainly TOP and IUFD led to the overall adverse outcome of 40% in our study. In most published series, termination was performed in 69–88% of pregnancies and miscarriages/IUFD occurred in 12–27% [4, 5, 18–20].

There are very few reported cases of VAS before 14 weeks of gestation [13, 21]. Most publications, including the randomized PLUTO trial, evaluated treatment in the second trimester [2, 14, 22]. 2nd trimester VAS reduces pulmonary hypoplasia without having hardly any effect on long-term renal impairment [14, 15, 23]. Neonatal survival after second trimester VAS ranges from 32 to 57% in these studies [14–16, 22, 23]. The reason for the lack of impact on renal function in second trimester VAS might be that it is performed too late to salvage renal function. Animal studies have shown that obstructive uropathy leads to a reduction in nephrogenesis and there is evidence that longer duration of the obstruction worsens renal function and decompression might delay or even reverse this deterioration [6, 7, 24–26]. In human fetuses after TOP, renal dysplasia has not been shown to be present before 15 weeks [27], the reason why we decided to offer VAS before this gestational age.

Early VAS has so far been considered technically impossible prior to 16/17 weeks due to its associated high rates of miscarriage, PPROM and dislocation with the thicker previously used shunt systems. We have shown that the smaller Somatex^®^ shunt can be safely used from the late first trimester with a reduction of pulmonary mortality and a better chance to salvage renal function. Another advantage is the easy visualization of the Somatex shunt on ultrasound due to its wire mesh that also makes it more stable, while the parasols and the silicone membrane prevent dislocation.

Renal function at birth was considered normal in all our surviving neonates according to local standard by the treating neonatologist, but we have to assume severe renal insufficiency in the one neonate that died of pulmonary hypoplasia following anhydramnios. As this was a feasibility study of first trimester VAS, we have no data on long-term renal function and we know that there will be deterioration of renal function over time. The very sparse results of published early interventions however are promising with high rates of normal renal function even after several years [13, 21, 28].

### Shunt dislocation

Dislocation rates after second trimester VAS have been described from 40 to 66% using Harrison^®^ Fetal Bladder Stents (Cook Medical, USA). In our cohort, the dislocation rate was 40%, considerably lower than in the only other cohort study of early VAS (but after 14 weeks), with a dislocation rate of 100% [13]. Three dislocations occurred in the late second or third trimester after 12, 16, 17 weeks into the abdominal cavity of the fetus making a second shunt insertion necessary. These long intervals allowed maintenance of normal amounts of amniotic fluid during the crucial time of pulmonary development.

The main controversy surrounding fetal intervention in megacystis is appropriate candidate selection, which is even more difficult in the first trimester. A male fetus with isolated PUV is usually considered to be the best candidate for intrauterine treatment, but there are associated genetic or structural anomalies in about a third of fetuses with megacystis. Genetic testing prior to VAS should therefore be offered to every patient.

First trimester diagnosis of megacystis by itself is reported to be associated with a poorer prognosis [3–5, 17], as urethral stenosis/atresia has been reported to be equally common as posterior urethral valves (PUV) in series with an early diagnosis [17, 29, 30]. Considering only surviving neonates in our cohort, PUV was the underlying pathology in 50% of the boys, urethral stenosis was found in 33% and one boy had a very complex cloacal malformation, which had been suspected prenatally but the parents opted to continue the pregnancy. There is also evidence in the literature that in cohorts including both sexes megacystis-micocolon-hypoperistalsis-syndrome (MMHIS) and ARM/cloacal malformations might be more common when the diagnosis of megacystis is made earlier in pregnancy and associated with larger bladders at diagnosis [19].

Due to the retrospective design and very small number of our study there are limitations, especially the lack of information in cases of TOP and IUFD because most parents refused a post-mortem examination. We can therefore base our conclusions regarding etiology and outcome mainly on live-born neonates. In addition, there is no first trimester control group, as all patients in our centers choose either early VAS or termination of pregnancy after a first trimester diagnosis during the study period, but in the literature survival after first trimester megacystis > 15 mm is very low and it is sometimes even considered a lethal entity [17]. A general limitation is the inability to predict long-term renal outcome in the first trimester due to several inherent factors: oligo/anhydramnios is usually not present before 15/16 weeks, the kidneys almost always seem hyperechogenic on ultrasound and hydronephrosis may be only visible after shunt placement, corticomedullary differentiation is not distinguishable on ultrasound in the first trimester and due to rudimentary tubular function in the first trimester urine biochemistry is not likely to be predictive of renal function after birth. Even in the second trimester there is conflicting data of the value of urine biochemistry in predicting renal function after birth, but there is hardly any data on urine biochemistry before 14 weeks (neither in normal fetuses nor LUTO). The only study evaluating fetal urine biochemistry before 23 weeks in LUTO included only three cases before 14 weeks in which no parameter distinguished well between normal and abnormal renal function [31].

The question remains how to counsel parents when faced with a large megacystis in the first trimester. Is there an advantage of waiting for 16/17 weeks to perform VAS? In our opinion based on our results, the answer is no. If patients opt for fetal intervention after counselling about the limitations of VAS in general, the risk of associated malformations and lack of long-term outcome, VAS should be performed as early as possible, as renal function might deteriorate considerably even by waiting a couple of weeks. Using a Somatex shunt, the risk of complications and shunt dislocation is less or at least similar compared to second trimester shunting. There are no reported spontaneous resolutions in the literature of LBD > 15 mm in the first trimester [4, 5, 18] and optimal candidate selection for VAS will still be difficult even at 16 weeks. Repeated vesicocentesis, which is sometimes performed until 17 weeks due to lack of other options, comes at the expense of repeated interventions, therefore increasing the overall risk of complications. In addition, there is no data evaluating at which intervals this needs to be done to have a positive effect on long-term renal function.

## Conclusion

We have demonstrated that VAS in the first trimester using a Somatex^®^ shunt is technically feasible without increasing maternal or fetal complication rates. First trimester VAS increased neonatal survival considerably in our cohort and has the potential to retain renal function. Children that are live-born have a low rate of renal insufficiency at birth, but long-term follow-up of these children regarding renal and overall morbidity is still lacking. Further research is still needed and we are planning to include patients in a prospective cohort study for first trimester VAS to assess morbidity of co-existing anomalies as well as long-term renal and bladder function.
